# Development and optimization of a tamsulosin nanostructured lipid carrier loaded with saw palmetto oil and pumpkin seed oil for treatment of benign prostatic hyperplasia

**DOI:** 10.1080/10717544.2022.2105448

**Published:** 2022-08-01

**Authors:** Rana B. Bakhaidar, Khaled M. Hosny, Imman M. Mahier, Waleed Y. Rizq, Awaji Y. Safhi, Deena M. Bukhary, Muhammad H. Sultan, Haitham A. Bukhary, Osama A. Madkhali, Fahad Y. Sabei

**Affiliations:** a Department of Pharmaceutics, Faculty of Pharmacy, King Abdulaziz University, Jeddah, Saudi Arabia; b Department of Biotechnology, Cairo Clinical Laboratory Center, Cairo, Egypt; c Department of Pharmaceutics, College of Pharmacy, Jazan University, Jazan, Saudi Arabia; d Department of Pharmaceutics, College of Pharmacy, Umm Al-Qura University, Makkah, Saudi Arabia

**Keywords:** Tamsulosin, nanoparticle, drug delivery system, experimental design, prostate index

## Abstract

Benign prostatic hyperplasia (BPH) is a nonmalignant growth of the prostate tissue and causes urinary tract symptoms. To provide effective treatment, tamsulosin (TM), saw palmetto oil (SP), and pumpkin seed oil (PSO) were combined and fabricated a nanostructured lipid carrier (NLC) as TM-S/P-NLC using experimental design. The purpose was to enhance the permeation and therapeutic activity of TM; combining TM with SP and PSO in an NLC generates a synergistic activity. An optimized TM-S/P-NLC was obtained after statistical analysis, and it had a particle size, percentage of entrapment efficiency, and steady-state flux of 102 nm, 65%, and 4.5 μg/cm^2^.min, respectively. Additionally, the optimized TM-S/P-NLC had spherical particles with a more or less uniform size and a stability score of 95%, indicating a high level of stability. The in vitro release studies exhibited the optimized TM-S/P-NLC had the maximum release profile for TM (81 ± 4%) as compared to the TM-NLCs prepared without the addition of S/P oil (59 ± 3%) or the TM aqueous suspension (30 ± 5%). The plasma TM concentration–time profile for the TM-S/P-NLC and the marketed TM tablets indicated that when TM was supplied in a TM-S/P-NLC, the pharmacokinetic profile of the drug was improved. Simultaneously, in vivo therapeutic efficacy studies also showed favorable results for the TM-S/P-NLC in terms of the prostate weight and prostate index following treatment of BPH. Based on the findings of present study, we suggest that in the future, the TM-S/P-NLC could be a novel drug delivery system for treating BPH.

## 1. Introduction

Benign prostatic hyperplasia (BPH) is one of the nonmalignant growths of prostate tissue and leads to lower urinary tract symptoms (LUTS) in men. As per the published reports, the global burden of BPH increased from 5.48 million in 1990 to 11.26 million in 2019, and the cases are increasing exponentially (Lim, 2017). BPH results from histopathological changes in the prostate and causes enlargement of the benign prostate, increased glandular size, and outlet obstruction. BPH causes a proliferation of stromal and epithelial cells in the prostatic transition zone, leading to urethral compression, urinary retention, and incomplete bladder emptying (Roehrborn, 2005). Studies have also reported the overactivated role of PDE5 (phosphodiesterase type 5) in the urinary tract, a substance that modulates the myosin phosphate activity and contraction of prostrate muscles (Alhakamy et al., 2019). If BPH is poorly managed or remains untreated, a potentially life-threatening emergency can occur and can be fatal.

Currently, the treatment options for BPH are pharmacotherapy and surgical interventions. The pharmacotherapies used are α-blockers such as tamsulosin (TM), alfuzosin, doxazosin, or terazosin; 5α-reductase inhibitors such as finasteride and dutasteride; and antimuscarinic drugs such as tolterodine, solifenacin, oxybutynin, and mirabegron. TM is extensively used either alone or in combination with others of these drugs. TM is the drug of choice for BPH or LUTS and acts via a blockade of the adrenergic receptors leading to the relaxation of prostatic smooth muscles and improvement in the overall symptoms (Skinder et al., 2016). TM offers a 20 to 38 times higher affinity for the α1A receptors than the α1B receptors and is used by more than 80% of clinicians to manage and treat BPH (Narayan & Tunuguntla, 2005). Various clinical trials have established the clinical efficacy of TM when used at doses of 0.4 mg and 0.8 mg (Lepor, 1998; Narayan & Tewari, 1998). Despite the significant clinical outcome of TM, its use is limited by pharmacodynamic and pharmacokinetic limitations. As proven, inflammation is one of the critical pathological contributors in the etiology of BPH, and TM is devoid of any anti-inflammatory effect. Also, the use of TM is associated with a negative impact on sexual life by causing priapism (Khater et al., 2020).

As researchers considered the pharmacokinetic profile of TM, it was found that TM possesses a variable absorption profile, and this could be one of the reasons for its adverse effects and reduced clinical outcomes (Lyseng-Williamson et al., 2002). It has also been reported that the available TM formulation undergoes a drug release phase in the presence of water, and hence a higher drug release profile is seen in the stomach and intestine (Chapple & Chartier-Kastler, 2006). It has been reported that after oral administration, TM increases the risk of postural hypertension, dizziness, orthostatic hypotension, and syncope (Michel et al., 2005). These limitations of TM may cumulatively lead to poor clinical outcomes and a reduced quality of life for patients. Hence, the development of a novel type of drug delivery and a new formulation of TM is needed for the drug to have an effective therapeutic value.

Pumpkin seed oil (PSO) belongs to the Cucurbitaceous family. It exhibits potent activity against nocturia and urinary incontinence and reduces the frequency of urination (Roehrborn et al., 2015). It was also found that the use of a hydroalcoholic extract of pumpkin seeds exhibited a protective effect against hyperplastic and cancerous cells, and the findings strengthened the antihyperplastic role of PSO (Leibbrand et al., 2019). Studies have also shown that PSO exhibited a protective effect against testosterone-induced prostate hyperplasia via the inhibition of 5-α-reductase (Vahlensieck et al., 2015). The chemical composition of PSO includes phytosterol, fatty acids (oleic acids, palmitic acid, linoleic acid), triterpene, and tocopherols (Nawirska-Olszańska et al., 2013). In the Caribbean region, P is used as a nutraceutical and dietary supplement, whereas in North India, Mexico, North America, Europe, and China, its use for bladder disease is gaining acceptance (Alhakamy et al., 2019). Thus, based on published reports, it was found that PSO could be an emerging treatment modality for the management and treatment of BPH.

Apart from the proven role of PSO in BPH, the SP, or *Serenoa repens*, a palm that is found in the south coastal region of the United States, has also been extensively studied for treating BPH. Native Americans use it for various urinary and prostate problems. SP consists of various phytosterols, fatty acids, and vitamin E and acts via inhibiting 5α-reductase, cyclooxygenase, and 5-lipoxygenase. Additionally, SP has an antiproliferative effect against epithelial cells of the prostate (Governa et al., 2016). SP has been studied alone and in combination with other alpha-blockers and 5α-reductase inhibitors in clinical settings. In a multicentric trial, when SP extract was used for six months, marked improvement occurred in the International Prostate Symptom (IPSS) score and flow of urine (Giulianelli et al., 2012). Similarly, SP also showed long-term efficacy and safety against LUTS (Sinescu et al., 2011; Ye et al., 2019). Furthermore, based on Braeckman’s study, the use of SP oil for three months at a dose of 160 mg before bedtime (BD) improved the IPSS score to 22% and 35% after 45 and 90 days, respectively (Braeckman, 1994). In another study by Hong et al. (2009), the use of the combination of P and SP for 12 months in 47 Korean men significantly improved the IPSS score, improved the quality of life, reduced the serum prostate-specific antigen, improved the urinary flow rate.

Nanostructured lipid carriers (NLCs) are newer-generation lipidic nanoparticles introduced by Muller et al. as an alternative to solid lipid nanoparticles. NLCs offer the advantage of increased entrapment efficacy, more sustained drug release, and maintenance of the physicochemical stability of drugs, and they possess higher bioavailability and biocompatibility (Iqubal et al., 2020; Costa et al., 2021). NLCs are spherical in shape, with a mean diameter ranging from 50 to 500 nm; they are prepared by combining solid lipids and oils, which are dispersed in the aqueous phase and stabilized by using surfactants. Oils used in the fabrication of NLCs help in the formation of an amorphous nanostructure and are involved in multiple interactions inside the matrix (Iqubal et al., 2021). Moreover, NLCs are considered safe because they use biodegradable carriers and their ingredients are generally recognized as safe. Therefore, to obtain an optimized formula for the preparation of a TM-loaded NLC of SP and PSO (TM-S/P-NLC), experimental design software (Ver. 11.1.1.0; Stat-Ease, Inc., Minneapolis, MN, USA) was used to generate a three factors-based, face-centered central composite design (CCD) (Imran et al., 2020).

It is further known that as of now, only TM nanotransfersomes and microemulsion of TM and dutasteride have been developed. Moreover, a combination of SP–TM was tested in an open-label, randomized study, whereas PSO–SP oil was tested in Korean men with symptomatic BPH. Moreover, these published reports have not been evaluated in the in vivo studies.

Thus, keeping in line with these published reports, we have first time fabricated the drug and natural products combination, i.e. TM, SP oil, and PSO for the treatment of BPH. Additionally, this is the first report of TM-NLC loaded with SP and PSO (TM-S/P-NLC) to treat BPH and evaluate the success of formulation in the in vivo study.

Hence, a novel formulation was fabricated to enhance the permeation and therapeutic activity of TM in treating BPH; this would occur via the synergistic activity of TM when given with SP and PSO as an NLC. The therapeutic activity could be obtained through the improved antioxidant, anti-inflammatory, and 5α-reductase inhibitory potential, which could synergistically potentiate the effect of TM via the inhibition of α-adrenergic receptor-mediated smooth muscle contraction. Additionally, the NLC offered the advantages of the incorporation of oils, the provision of sustained drug release, and eventual improved efficacy against BPH.

Based on the aforementioned facts, the aim of the current work was to formulate TM in the form of NLC combined with SP and PSO oils and deliver it systemically via transdermal route to avoid the major side effect associated with TM which is its variable absorption from GIT.

Thus, fabrication of NLC drug and natural products combination and evaluation of the success of this formulation in the in vivo study is the novelty of this study. In other words, an optimized TM-S/P-NLC was prepared using CCD to characterize selected parameters such as particle size, percentage of entrapment efficiency (EE), and ex vivo steady-state flux (Jss). The optimized nanoformulation was then evaluated for its nanomorphological characteristics, stability, drug release capabilities, pharmacokinetic behavior, and in vivo efficacy against BPH.

## 2. Materials and methods

### 2.1. Materials

TM was acquired as a generous gift from the Saudi Arabian Japanese Pharmaceutical Company Limited (Jeddah, Saudi Arabia). SP and PSO oils were procured from Acros Organics (New Jersey, USA). Glyceryl distearate (Precirol ATO5), caprylocaproyl polyoxyl-8 glycerides (Labrasol), and propylene glycol monocaprylate (Capryol 90) were obtained from Gattefosse (Saint-Priest, France). High-performance liquid chromatography-grade methanol, chloroform, and acetonitrile were obtained from Merck (Darmstadt, Germany). Absolute ethanol and phosphate buffer at pH 7.4 were purchased from Fisher Scientific UK (Loughborough, Leicestershire, UK). All other reagents and chemicals used were of analytical grade.

### 2.2. Experimental design

For the development and selection of the optimized TM-S/P-NLC nanoformulation, an experimental design-based CCD was applied using Design Expert software (Hosny et al., 2020; Imran et al., 2020). The effects of the three independent formulation factors, namely, the lipid-to-TM ratio (A), S/P-to-Precirol ratio (B), and Labrasol concentration (C), on the prepared NLCs particle size (PS), percentage of EE, and Jss, respectively, were analyzed (Table 1). This CCD experimental design produced 19 runs of different concentrations of selected independent factors (Table 2).

**Table 1.** The variables employed to select an optimized TM-S/P-loaded NLC using the CCD.

Factors	Levels		
Independent variables	−1	0	+1
A: Lipid-to-TM ratio	10:1	25:1	50:1
B: S/P-to-Precirol ratio	1:9	2:8	3:7
C: Labrasol (%)	1	2	3
Dependent variables	Goal		
R_1_: Particle size (nm)	Minimum		
R_2_: Percent entrapment efficiency	Maximum		
R_3_: Ex vivo steady-state flux (Jss, μg/cm^2^.min)	Maximum		

**Table 2.** Outcomes of CCD for the preparation and optimization of TM-S/P-NLC nanoformulations.

Run	Factor A	Factor B	Factor C	Response 1	Response 2	Response 3	Zeta Potential (mV)	PDI
	Lipid-to-TM ratio	S/P–Precirol ratio	Labrasol (%)	Particle size (nm)	EE (%)	Steady-state flow (μg/cm^2^.min)		
1	−1	1	1	66	38	5.4	−23.4	0.315
2	0	0	−1.682	176	89	3.2	−22.6	0.411
3	−1	−1	1	70	41	3	−24.4	0.377
4	1	−1	−1	210	94	2.1	−25.2	0.411
5	0	0	0	140	66	3.6	−21.2	0.362
6	−1	−1	−1	102	65	2.2	−26.3	0.298
7	−1.682	0	0	54	52	3.7	−23.1	0.331
8	1.682	0	0	210	79	4	−24.2	0.313
9	0	−1.682	0	145	68	1.7	−22.7	0.354
10	0	0	0	135	61	3.8	−21.9	0.411
11	0	0	0	143	67	4.1	−21.6	0.338
12	0	1.682	0	138	63	5.9	−23.3	0.409
13	1	−1	1	170	67	3.1	−25.3	0.399
14	1	1	−1	197	91	4.1	−25.7	0.298
15	0	0	0	144	68	4	−24.8	0.401
16	1	1	1	165	65	5.6	−25.5	0.466
17	0	0	1.682	110	49	4.8	−23.3	0.391
18	0	0	0	145	70	4.2	−22.6	0.406
19	−1	1	−1	99	64	4.4	−21.5	0.294

### 2.3. Preparation of TM-S/P-loaded NLCs

First the solubility of TM in SP and PSO oils was checked by dissolving excess amount of TM in SP and in PSO oils and then were placed for 72 h in a water bath instrument at 25 ± 2 °C (Model 1031; GFL Corporation, Germany). Following equilibrium, the rested mixtures were then centrifuged (Sigma 3k30, Germany) at 4500 rpm for 15 min. Then, the supernatants were separated and diluted with methanol and TM concentration was estimated at 225 nm λ_max_ using a reported High-performance liquid chromatography (HPLC) method. For the preparation of TM-S/P-loaded NLCs hot high shear homogenization method was used. A hot high shear homogenizer is generally used to break the larger particles into smaller particles. But nanosized particles are difficult to obtain through this process, so for the preparation of NLC dual regime is used (Yukuyama et al., 2016; Mura et al., 2021). Thus, different formulations of TM-S/P-NLCs were prepared using a hot high shear homogenization method followed by a sonication technique (Baek et al., 2015; Motawea et al., 2018). Each formula contained an oil phase, composed of predetermined quantities of Precirol ATO5 according to the design (100, 250, or 500 mg); S/P mixture in the ratio of 1:9, 2:8, or 3:7, depending on the total lipid content; Capryol-90 10 mg; and TM 10 mg dissolved in a 1:1 mixture of chloroform and methanol. A rotary evaporator (R 10 BÜCHI Labortechnik AG, Büchi, Flawil, Switzerland) was used to remove the organic solvents completely. The drug-embedded lipid layer was melted by heating it at 60 to 65 °C. The aqueous phase was prepared by adding predetermined quantities of Labrasol (1%, 2%, and 3%) in distilled water and heating the mixture to the same temperature as the oil phase (i.e. 60 to 65 °C). The warmed aqueous phase was added to the oil phase, and the mixtures were homogenized using IKA homogenizer T18 (at a speed of 6,000 to 24,000 rpm) for 2 min (IKA, Wilmington, NC, USA). The prepared emulsions phase was sonicated for 3 min using a probe sonicator (Sonics Vibra-Cell VCX 750, Sonics & Materials, Inc., Newtown, CT, USA) to form the NLCs, and it was left to cool at room temperature (i.e. 25 °C). The NLCs were then lyophilized (Martin Christ GmbH, Osterode am Harz, Germany) with a condenser temperature of −45 °C and pressure of 7 × 10^−2^ mbar (Rahman et al., 2013); 20 ml of nanoformulations were subjected to lyophilization, and then subjected to characterization studies.

### 2.4. Characterization of prepared TM-S/P-NLCs

#### 2.4.1. Particle size, zeta potential, and polydispersity index determination

One hundred milligrams of each prepared NLC formulation was dispersed in 1 ml of double-distilled water, and then 100 µl from each NLC dispersion was withdrawn. This amount was used to determine the globule size, zeta potential, and polydispersity index determination by dynamic light scattering using a Zetatrac particle size analyzer from Microtrac, Inc. (Montgomeryville, PA, USA)

#### 2.4.2. Determination of EE and loading efficiency (LE)

The EE was determined after centrifuging 1 ml of the TM-S/P-NLC in pH 7.4 phosphate buffer saline (PBS) with 15,000 rpm at 4 °C for 1 hour (Almousallam et al., 2015; Martinelli et al., 2020). The supernatant was separated from the centrifuged sample. The amount of free drug in the supernatant was determined by a reported HPLC method in which TM was chromatographed using a reverse-phase C_18_ column with a mobile phase consisting of acetonitrile:water in the ratio of 50:50 v/v. The mobile phase was pumped at a flow rate of 1.5 ml/min. The eluents were monitored at 214 nm. The retention time of the drug was 1.7 min (Kumari et al., 2010; Shah et al., 2022). The EE and LE were calculated using the following equations from the values of the free drug and the total amount of drug added (Tan et al., 2022).

(1) $$\mathrm{EE}\;(\%)=\frac{\text{Total amount of drug }–\text{ Free drug amount }}{\text{Total amount of drug }}\times 100$$

(2) $$\mathrm{LE}\;(\%)=\frac{\text{Total amount of drug }–\text{ Free drug amount }}{\text{Total amount of lipid added }}\times 100$$

#### 2.4.3. Determination of Jss

Ex vivo permeation of TM from the different TM-S/P-NLC formulations was studied using an automated Franz diffusion cell apparatus (MicroettePlus; Hanson Research, Chatsworth, CA, USA), which has a surface area of 1.76 cm^2^. Full-thickness excised abdominal male Wistar rat skin (2.5 × 2.5 cm^2^) was used as a membrane. The subcutaneous fat was removed from the excised rat skin and washed with Ringer solution before the experiment. The excised and washed rat skin was placed between the donor and receptor compartments of the Franz diffusion cell. The stratum corneum layer of the excised rat skin was kept facing toward the donor compartment of the Franz diffusion cell. Phosphate buffer pH 7.4 at 37 ± 0.5 °C was chosen as the medium for ex vivo permeation studies. The TM-S/P-NLC samples were placed in the donor compartment. Aliquot samples from the receptor medium were withdrawn at time points of 1, 2, 4, 6, 8, 10, 12, and 24 hours after placing the TM-S/P-NLC samples in the donor compartment. During each sampling, fresh buffer previously heated to 37 ± 0.5 °C was added to make up for the removed volume of the receptor medium (Assaf et al., 2019; Almehmady & Elsisi, 2020). The TM concentrations in the withdrawn samples were then determined using a previously mentioned HPLC method (Kumari et al., 2010). The steady-state flux of the TM was determined from the concentration values for each sample.

### 2.5. Evaluation of optimized TM-S/P-NLC

#### 2.5.1. Determination of zeta potential

The zeta potential analysis was performed on a Zetatrac particle size analyzer (Microtrac, Inc., Montgomeryville, PA, USA). For this purpose, conductivity was corrected to 50 mS per cm using 0.9% w/v sodium chloride before analysis, and double-distilled water was utilized right through the process. The collected sample of optimized nanoformulation was diluted to 10-folds with double-distilled water and transferred to a cuvette for zeta potential determination, and the process was performed in triplicate at 25 ± 2 °C (Iqubal et al., 2021).

#### 2.5.2. Morphological examination

The nanomorphological characteristics of the optimized TM-S/P-NLC formulation were studied by transmission electron microscopy (TEM, H7500, Hitachi, Japan). In this case, 1 drop of the optimized nanoformulation was dried on a copper grid, and the dried sample was stained with uranyl acetate (2% w/w). Further, the stained sample was air-dried to eliminate excess liquid medium and finally analyzed under the TEM (Jangdey et al., 2017).

#### 2.5.3. Stability study

To analyze the stability of the optimized TM-S/P-NLC, thermodynamic investigations were conducted using three freeze-thaw cycles. Each cycle consisted of 12 hours of freezing at –25 °C followed by 12 hours of thawing at +25 °C. To ensure the stability of the TM-S/P-NLC, the final nanoformulation was tested for changes in particle size and zeta potential. The equation used to calculate the stability index of the NLC nanoformulation is as follows (Almehmady & Elsisi, 2020):

(3) $$\text{Stability index}=\frac{\text{Initial size }–\text{ Change in size}}{\text{Initial size}}\times 100$$

#### 2.5.4. *In vitro* drug release

This experiment used a USP dissolution apparatus I. Here, instead of baskets, glass cylinder tubes with a diameter of 2.7 cm and a length of 10 cm containing the tested formulation were attached to the spinning shafts and tightly coated with semipermeable membranes (cutoff 14,000 Da pore size) (Hosny et al., 2021). The amount of TM (0.4 mg) in the 1 ml of each formulation (optimized TM-S/P-NLC, TM-NLC made without the addition of S/P oil, and TM aqueous dispersion) was determined in the glass tubes. The tubes were put into 50 mL of phosphate buffer (pH 7.4) solution, which contained 0.1% poloxamer to maintain the sink condition of the drug release medium. The medium was agitated at 25 rpm and ensured that the environmental temperature was at 37 ± 0.5 °C. Further, samples were taken out of the release media at varying intervals of time (0.25, 0.5, 1, 1.5, 2, 3, 4, 6, 8, and 12 hours). The released amount of TM was analyzed using the previously mentioned HPLC method (Kumari et al., 2010). In addition, five kinetic models (zero-order, first-order, Higuchi, and Korsmeyer–Peppas functions) were used to fit the experimental data obtained from drug release studies.

#### 2.5.5. *In vivo* pharmacokinetic evaluation

The in vivo drug pharmacokinetic study was performed according to the institutional guidelines of the Animal Ethics Committee of Beni Suef Laboratory for animal studies, Beni Suef, Egypt, Approval No. (22/3-001-21). Twelve albino male rabbits weighing between 2 and 2.5 kg were used in this in vivo study. The rabbits were grouped into two groups, each containing six rabbits. In this experiment, the TM tablets as a reference were given to animals of group I at a dose of 0.04 mg/kg. At the same time, group II animals received a 0.04 mg/kg dosage of the optimized TM-S/P-NLC nanoformulation by transdermal application using transdermal system designed as small cylindrical cup with dimension (1 cm diameter and 1 mm height) with adhesive margin, this designed system filled with the tested formulation and adhere on the skin facing the formula to the skin directly. Blood was taken at predefined intervals of 0.25, 0.5, 1, 1.5, 2, 3, 4, 6, 8, and 12 hours, and the concentrations of TM in plasma were measured. The recorded data were used to calculate the T_max_, C_max_, AUC_0–inf_, k, t_1/2_, and relative bioavailability (Aljaeid & Hosny, 2016; Almehmady & Elsisi, 2020; Hosny et al., 2020).

#### 2.5.6. *In vivo* study for the determination of prostate weight and prostate index in rats

In this experiment, six groups of animals were used, and each group had eight rats. The study was performed according to the institutional guidelines of the Animal Ethics Committee of Beni Suef Laboratory for animal studies, Beni Suef, Egypt, Approval No. (13/5-004-21). The animals of Group A was not treated with testosterone or any therapy, and this group was labeled as the normal control group. For BPH induction, Group B was given testosterone enanthate subcutaneously (SC) 5 days a week for 2 weeks at a dose of 3 mg/kg body weight of the animal. Group C (TM-S/P-NLCs), Group D (TM-NLCs prepared without S/P oil), and Group E (S/P-NLCs prepared without TM) was co-administered with transdermal application of the samples, using transdermal system designed as small cylindrical cup with dimension (1 cm diameter and 1 mm height) with adhesive margin, this designed system filled with the tested formulation and adhere on the skin facing the formula to the skin directly, 1 hour before receiving 3 mg/kg testosterone enanthate SC 5 days a week for 2 weeks. In addition, Group F of commercially available TM tablet was co-administered with TM tablet, 1 hour before receiving 3 mg/kg testosterone enanthate SC 5 days a week for 2 weeks.

The rats were euthanized using CO_2_ euthanasia method. In brief, animals were placed into the chamber containing up to 70% CO_2_ where the rate of flow of gas was displace at least 20% of the chamber volume per minute. Additionally, a constant level of CO_2_ was maintained for at least 3 min and anesthesia occurred within 60 seconds followed by the euthanasia. Hence, 72 hours after receiving their final testosterone dose and euthanasia, their prostatic tissue was dissected. The rat prostates were removed and weighed as soon as possible. The prostate index was analyzed mathematically by dividing the prostate’s weight by the body weight (g/g) (Alhakamy et al., 2019).

### 2.6. Statistical analysis

The collected data were portrayed as the mean ± standard deviation (SD) after statistical analyses. A comparison of means was performed using a one-way analysis of variance (ANOVA) followed by a Tukey post-hoc test. Differences at *p*-values below .05 were deemed statistically significant.

## 3. Result and discussion

### 3.1. Selection of optimized TM-S/P-NLC using CCD-based experimental design

The results of solubility studies indicated that solubility of TM in SP and in SPO were found to be 57 mg/ml and 116 mg/ml, respectively. The influence of the independent parameters on the particle size, EE, and steady-state flow of the drug was calculated and statistically analyzed using ANOVA on the runs produced by the experimental design software. The analysis findings revealed that the obtained model was significant for all three dependent variables and that the suggested models fit well. Table 2 shows several model values for the observed dependent variables, such as R^2^, SD, and coefficient of variation (%CV).

#### 3.1.1. Impact of the selected independent variable on particle size

**Figure 1.**
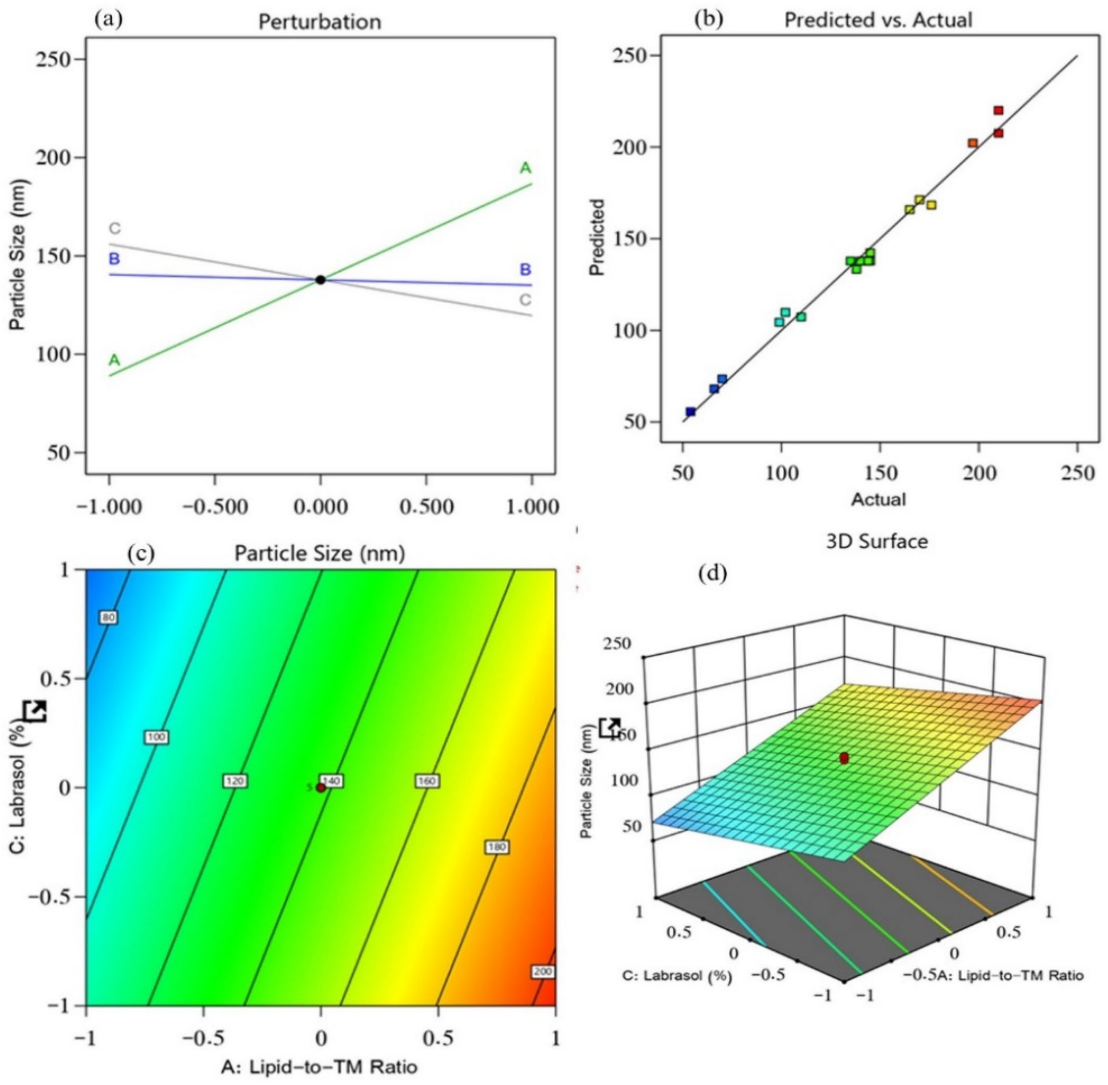
Effect of independent variables on the particle size of prepared NLCs: (a) main effect plot, (b) relationship between the actual and predicted R^2^ values, (c) contour plot, and (d) 3D surface plot.

In transdermal drug delivery, the rate of penetration and extent of drug distribution are mainly governed by the particle size. The ideal particle size required for improved bioavailability is less than 210 nm (Danaei et al., 2018). The difference between the predicted R^2^ value (0.9795) and the adjusted R^2^ value (0.9851) was found to be less than 0.2, implying a satisfactory agreement between the two (Figure 1a). The following equation was derived from the obtained model (*p* < .0001) with a model F-value of 397.02.

(4) Particle size = + 137.84 + 48.87 A − 2.69 B −18.16 C 

The obtained equation demonstrated the positive influence of factor A (lipid-to-TM ratio) on the particle size, which explained why the particle size of the prepared TM-S/P-NLCs was increased with an increased concentration of factor A. Factor B (S/P–Precirol ratio) and factor C (Labrasol) exhibited a negative impact on the particle size (Figure 1). This means that with an increased concentration of factors B and C, the particle size of the TM-S/P-NLCs decreased. Therefore, the outcomes clearly demonstrated the effect of oil on the particle size because the increased concentration of oil offers greater space for TM accommodation and hence ending up increasing the NLC size. Simultaneously, increased concentrations of Precirol and Labrasol decreased the particle size; this was due their amphiphilic nature, which exert its action through reducing the interfacial tension between the organic and aqueous phases and thus downsizing the produced NLC.

#### 3.1.2. Impact of the selected independent variable on EE

**Figure 2.**
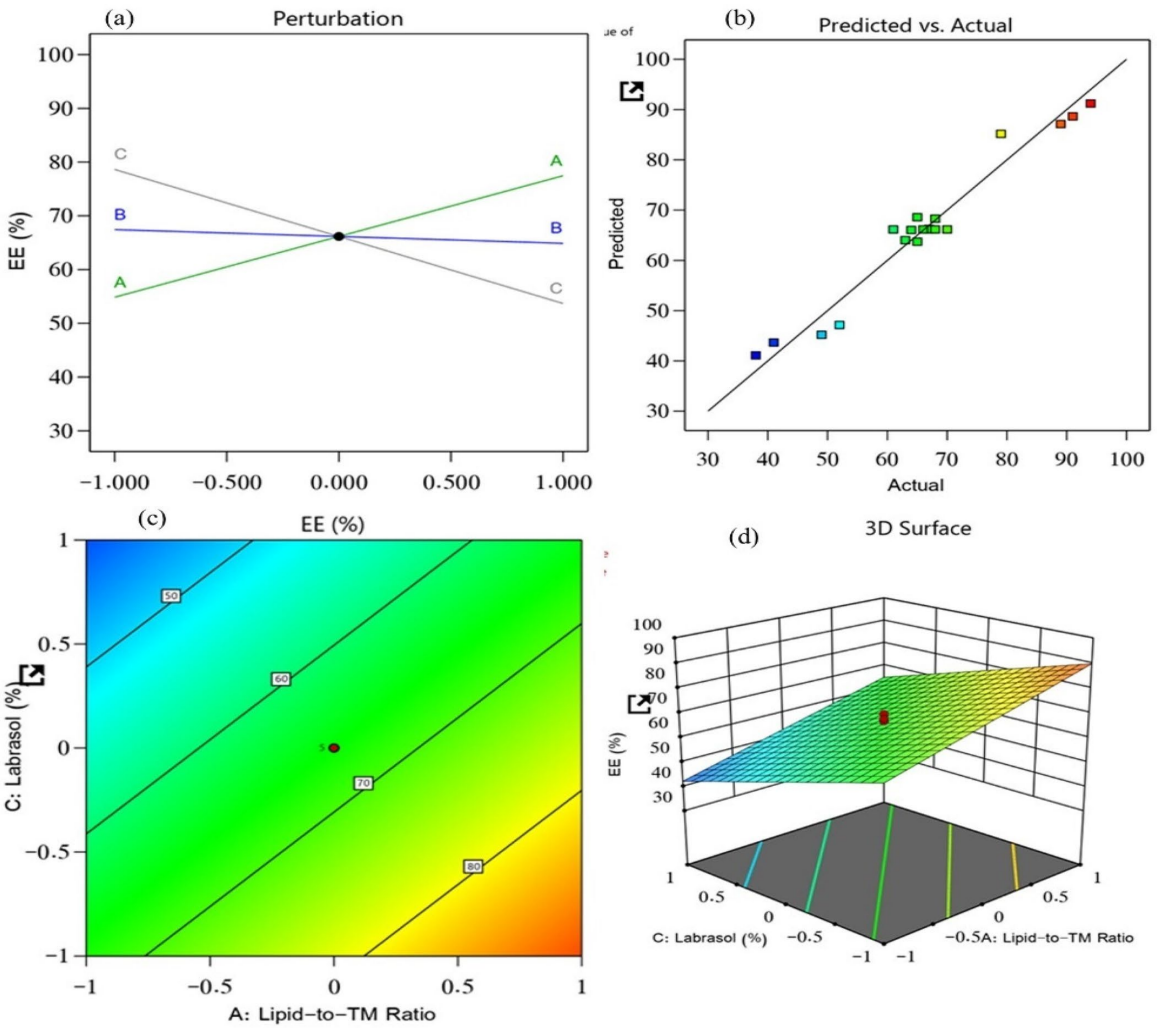
Impacts of independent variables on the EE of prepared NLCs: (a) main effect plot, (b) relationship between the actual and predicted R^2^ values, (c) contour plot, and (d) 3D surface plot.

The EE of the 19 prepared NLC nanoformulations was found to be between 38% and 94%. It is an essential metric that plays a key role in the formulation; it is dependent on the delivery of the dosage of the drug incorporated in the NLC that will provide clinical efficacy. The impact of the lipid-to-TM ratio, S/P–Precirol ratio, and surfactant (Labrasol) on this quality attribute was investigated. As a result, the generated model was used to fit the response data. The predicted R^2^ value (0.9284) and the adjusted R^2^ value (0.9482) were in reasonable agreement, with a difference of less than 0.2 (Figure 2). The following equation characterized the effect of the independent factors on the EE and recorded it from the obtained model (*p* < .0001) with a model F-value of 110.77.

(5) EE = +66.16 + 11.31 A − 1.27         B −12.47 C

A positive effect of the lipid-to-TM ratio was found for the EE of TM, whereas a substantial negative effect of the surfactant, as compared with the S/P–Precirol ratio, was found for the EE. The EE provided data about the amount of drug entrapped in the lipid, and it depended on the affinity of the drug with the lipid. Therefore, the lipophilic nature of TM is responsible for the increase in EE% upon increasing oil concentration. On the other hand, the surfactant, Labrasol, decreased the size of the lipid particles, thus provided smaller space for accommodating TM and also it increased the drug escape to the surrounding aqueous medium resulting in a decreased EE. The obtained model was found to be highly significant, as confirmed by the ANOVA analysis (*p* < .0001).

#### 3.1.3. Impact of the selected independent variable on steady-state flux

**Figure 3.**
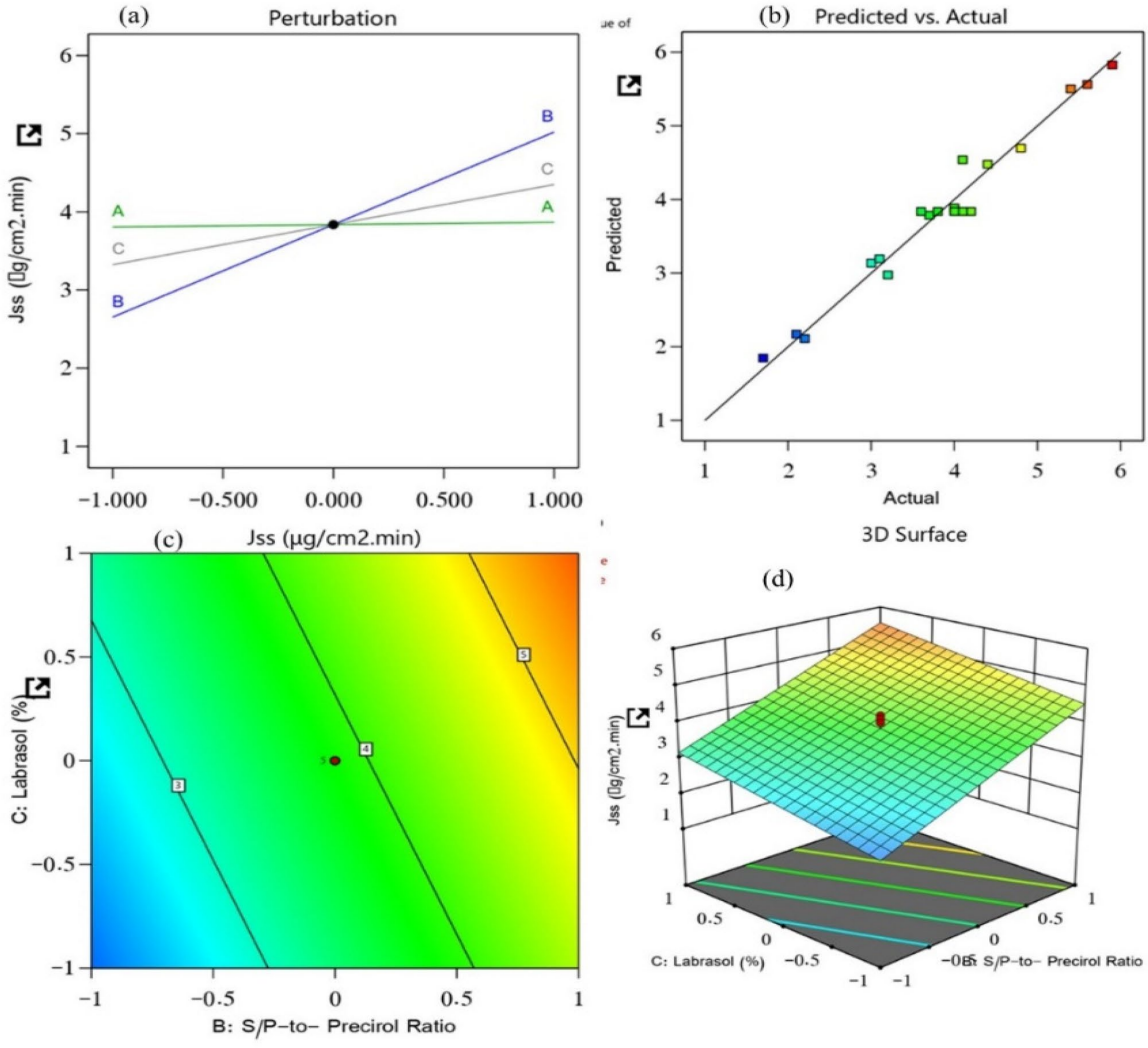
Effect of independent variables on the steady-state flux of prepared NLCs: (a) main effect plot, (b) relationship between the actual and predicted R^2^ values, (c) contour plot, and (d) 3D surface plot.

The steady-state flux of the 19 prepared NLC nanoformulations was found to be between 1.7 and 5.9 μg/cm^2^.min. It is an essential parameter that plays a key role in the drug diffusion from the nanoformulation to the disease site; it is dependent on the lipid and the surfactant that are incorporated into the NLC. The impacts of the lipid-to-TM ratio, S/P–Precirol ratio, and surfactant on the steady-state flux of the prepared NLCs were investigated. As a result, the generated model was used to fit the response data. The predicted R^2^ value (0.9577) and the adjusted R^2^ value (0.9670) were in reasonable agreement, with a difference of less than 0.2 (Figure 3). The following equation characterized the effect of the independent factors on the steady-state flux and recorded it from the obtained model (*p* < .0001) with a model F-value of 176.58.

(6) Steady−state flux (Jss) = +3.84 + 0.0296 A + 1.18 B + 0.5119 C

Equation (5) revealed the significant positive effects of all three selected factors on the steady-state flux. The lipid-to-TM ratio, S/P–Precirol ratio, and surfactant Labrasol were the factors. With the increased concentrations of these factors, the steady-state flux of the NLCs was found to be elevated. Of these factors, the S/P–Precirol ratio had the most pronounced impact on the steady-state flux. This means that with an increased concentration of factor B, the drug permeation rate and flux were accelerated. So, these results indicate that the permeation was enhanced with the S/P–Percirol ratio. Such increase in permeation may be due to the additive effect between the used oil and the NLC as nanosized drug delivery system which offers greater surface area for drug permeation. The observed positive effect of Labrasol on the flux (Figure 3a) might be due to the edge activator capacity of the surfactant, which could improve the flexibility and ability of he prepared NLC to show accelerated permeation and flux. Moreover, their amphiphilic nature could have contributed to fluidizing the stratum corneum which is the major barrier against permeation.

#### 3.1.4. Selection of optimized TM-S/P-NLC

**Figure 4.**
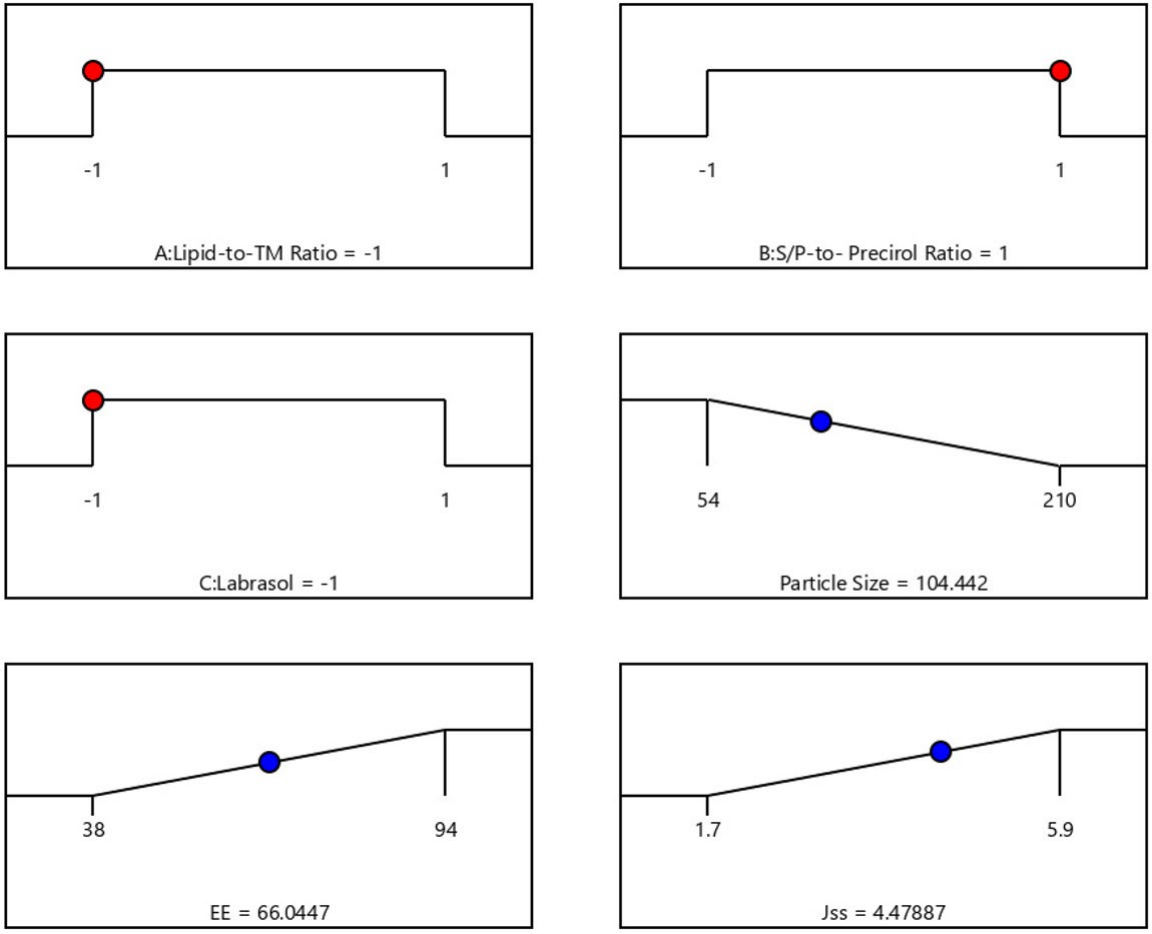
Desirability ramp showing the levels of the independent variables and predicted values for the responses of the optimized TM-S/P-NLC.

After completing various selected characterizations, the optimal TM-S/P-NLC was developed. Based on the runs generated by the Design Expert software (Table 2), different NLC nanoformulations were prepared with different ratios of the selected factors. After the response surface analysis, it was found that the optimized TM-S/P-NLC had a lipid-to-TM ratio of –1.0 (10:1), S/P–Precirol ratio of 1.0 (3:7), and Labrasol content of −1.0 (1%). The optimal nanoformulation had an observed particle size, zeta potential, PDI, EE, and flux of 102.01 ± 5.09 nm, −23.6 ± 2.11 mV, 0.311 ± 0.101, 65 ± 2.01%, and 4.50 ± 0.39 μg/cm^2^.min, respectively (Table 3), with a desirability equal to 0.9. This results indicated that the observed values for the tested parameters as particle size, EE, and steady-state flux were extremely close in agreement and had no significant differences (*p* > .05) from the predicted values obtained from the experimental design software, confirming the equation’s precision and validity for that optimization. The levels of the independent and expected values of the obtained responses of the optimized NLC nanoformulation are displayed in the desirability ramp in Figure 4. The observed and predicted values show that the optimized nanoformulation’s parameters were extremely close in agreement and had no significant differences (*p* > .05), confirming the equation’s precision and validity (Table 3).

**Table 3.** The experimental and predicted responses of the optimized TM-S/P-NLC.

Values	Lipid-to-TM ratio	S/P–Precirol ratio	Labrasol (%)	Particle size (nm)	Entrapment efficiency (%)	Steady-state flux (μg/cm^2^.min)	Desirability
Predicted value	10:1	3:7	1	104.44	66.04	4.48	0.908
Experimental value	10:1	3:7	1	102.01 ± 5.09	65 ± 2.01	4.50 ± 0.39	0.908

### 3.2. Morphologic examination of optimized TM-S/P-NLC

**Figure 5.**
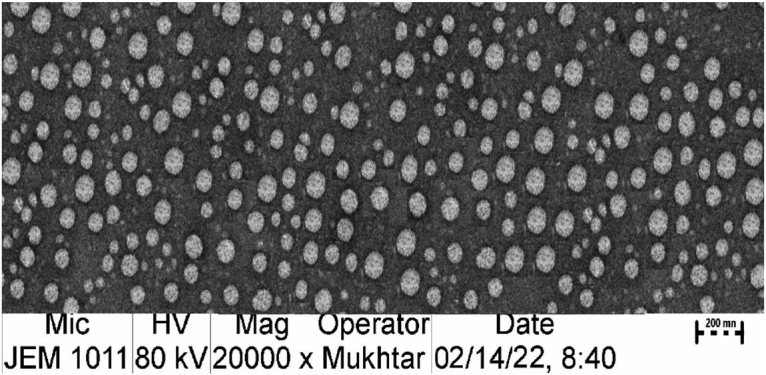
TEM image of optimized TM-S/P-NLC.

Figure 5 shows that the TEM-operated nanomorphological examination of the optimized TM-S/P-NLC revealed spherical particles with a more or less uniform size. The TEM image also showed that the size of the individual NLC particles was less than 200 nm; this agreed well with the results of the particle size examination.

### 3.3. Stability study

The stability of the TM-S/P-NLCs was revealed by thermodynamic experiments. Freeze-thaw cycles were used to verify the thermodynamic stability of the optimized TM-S/P-NLC. The initial particle size of optimized nanoformulation was 102 ± 2 nm, and after the freeze-thaw cycle, it was found 107 ± 2 nm. Simultaneously insignificant (*p >* .05) change in the zeta potential of optimized nanoformulation was obtained before (−23.6 ± 2.7 mV) and after the (−20.9 ± 1.8 mV) freeze-thaw cycle. With these outcomes, the obtained stability score was found to be 95%. So, it can be stated that the optimized nanoformulation had a high level of stability because the stability index has a maximum value of 100%, and a higher value indicates more excellent formulation stability. Thus, the optimized TM-S/P-NLC was found to be stable in the given thermodynamic conditions.

### 3.4. *In vitro* drug release

**Figure 6.**
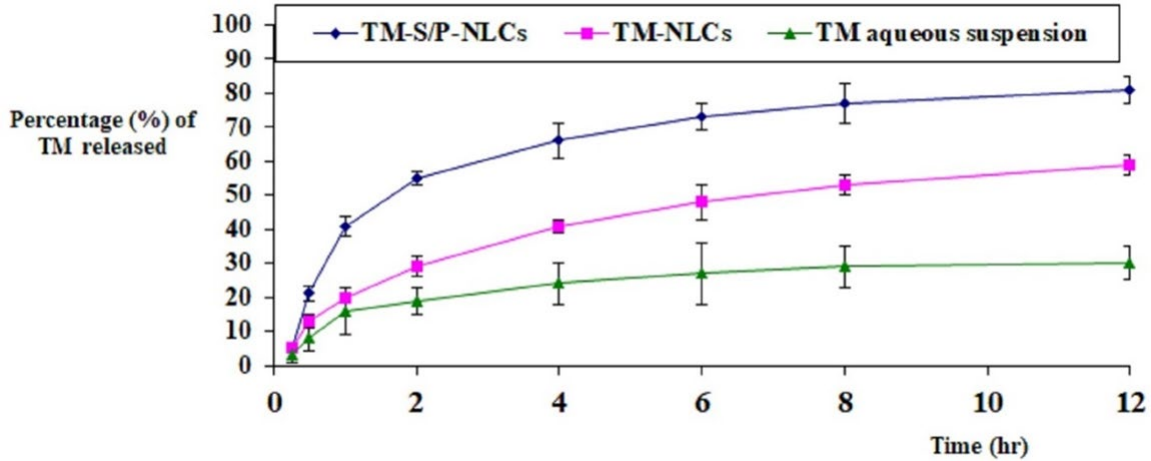
Comparative in vitro release profile between TM-S/P-NLCs, TM-NLCs, and TM aqueous suspension.

The comparative in vitro release studies demonstrated that the optimized TM-S/P-NLC had the maximum release of TM (81 ± 4%); it was more significant (*p <* .05) compared to drug release from the TM-NLCs prepared without the addition of S/P oil (59 ± 3%) or TM aqueous dispersion (30 ± 5%), as shown in Figure 6. The increase in TM release from TM-S/P-NLCs could be attributed to the effect of formulating the drug in the form of NLC in which the drug present in nanosize range compared to conventional TM aqueous suspension whose size falls in the coarse dispersion particle size range which might be due to low aqueous solubility of the drug. In addition, the inclusion of the S/P oil could have increased the release of drug from NLC as the oils enhanced the dispersion of the drug within the NLC (Mendes et al., 2013; Qumber et al., 2021). Such phenomena could be understood in the light of the facts implying that oil is responsible for the organized structure of NLC, and it is well established that well-organized NLC shows better release than less-organized NLC (Teeranachaideekul et al., 2020). Thus, the selection of oil is a vital factor for the release of drug from prepared NLC, and in this case, S/P oil loaded NLC showed the effect of oil on release pattern than TM-NLCs, which exhibited better release than TM-NLCs and TM aqueous suspension. The TM-S/P-NLC also had a dual-release characteristic, with a quick initial release followed by a delayed release. The presence of the TM on the surface of the NLC may have caused the initial burst, and the integration of TM into the solid lipid core matrix of the NLC nanoformulation caused the slow release.

About the release kinetic, the process is dominated by anomalous diffusion (0.54 < *n* < 0.82); therefore, the release of TM from NLCs follow Korsmeyer–Peppars non-Fickian release model.

### 3.5. *In vivo* pharmacokinetic evaluation

To evaluate the pharmacokinetic behavior of the TM-S/P-NLC, a comparative in vivo study was carried out. In this study, the pharmacokinetic pattern of the TM-S/P-NLC was compared with that of a commercial tablet of TM. The selected animals were treated with TM in a dose of 0.04 mg/kg. The pharmacokinetic parameters used to estimate bioavailability were the T_max_, C_max_, AUC_0–inf_, k, t_1/2_, and relative bioavailability (Table 4). With the transdermal delivery of the optimized TM-S/P-NLC, a significant (*p <* .05) increase in the C_max_ and AUC_0–inf_ was found. When compared with the tablet formulation, the C_max_ for the optimized nanoformulation was 2.73 times greater. For the TM-S/P-NLC, the AUC_0–inf_ of TM was increased by 3.02 times. Transdermal nanoformulations had already been shown to improve the bioavailability as compared with other drug delivery systems (Morsi et al., 2016; Pangeni et al., 2016).

**Table 4.** Pharmacokinetic parameters of TM from various sample formulations.

PK parameters	TM-marketed tablets	Optimized TM-S/P-NLC dispersion
C_max_ (ng/ml)	60.11 ± 6.1	164.22 ± 17.21
T_max_ (h)	1.5 ± 0.5	2 ± 0.5
t_1/2_ (h)	1.33 ± 0.32	2.59 ± 0.44
AUC_0-inf_ (ng/ml.h)	991.11 ± 89.27	2993.77 ± 201.14
K_el_ (h^−1^)	0.53 ± 0.04	0.267 ± 0.02
Relative BA (%)	**–**	3.02-fold

**Figure 7.**
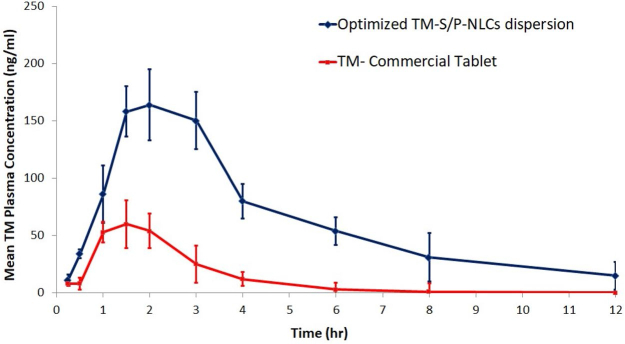
Plasma TM concentration–time profile for the TM-S/P-NLC and marketed tablets.

When TM was given as a TM-S/P-NLC, its T_max_ was increased. The T_max_ of tablets of TM was 1.5 hours, but the T_max_ of the TM-S/P-NLC was enhanced to 2 hours. Nonetheless, the TM-S/P-NLC had a greater plasma TM concentration at 1.5 hours. As a result, there is no evidence that the nanoformulation was able to improve the TM bioavailability. The time it took to reach a steady state of TM transport across the skin barriers could have shifted the role of the T_max_. The TM-S/P-NLC depot action could possibly play a role in such behavior. The delayed T_max_ showed that TM was released from the TM-S/P-NLC in a sustained or controlled manner. Meanwhile, the high lipophilicity of the TM-S/P-NLC may have contributed to the increased absorption capacity. The drug’s t_1/2_ and k were found to vary depending on the formulation’s behavior. Figure 7 shows the plasma TM concentration–time profile for the TM-S/P-NLC and marketed TM tablets. Overall, it was clear that when TM was supplied in a TM-S/P-NLC, the pharmacokinetic profile of the drug was improved. This is certainly related to the TM-S/P-NLC’s ultradeformability. It quickly squeezed through pores in the membrane and transported the drug to the systemic circulation. When comparing the TM-S/P-NLC with the tablet, the AUC was clearly greater in case of the nanoformulation.

The plasma TM concentration–time profile shows that the TM was completely eliminated from the body in approximately 12 hours for the TM-S/P-NLC and 6 hours for the tablets. Both the absorption and elimination phases of the TM-S/P-NLC were characterized by perpendicular curves. The nanoformulation had higher variability in individual values, as evidenced by the SD bars. Variability could be caused by changes in the skin’s structure or the site of application on the skin. However, as compared with the marketed tablets, the increase in bioavailability was considerable.

### 3.6. *In vivo* study for the determination of prostate weight and prostate index in rats

As shown in Table 5, the group with the testosterone challenge had a significantly (*p* < .05) increased prostate weight (by 60.56%) and prostate index (by 55.42%) compared with the control group. A significant (*p* < .05) decrease in the prostate weight and prostate index was noted in the treatment groups using the transdermal application of the optimized TM-NLC without S/P oil and the optimized S/P-NLC without TM compared with the testosterone-only group. This means that both components of the optimized formulation (TM and S/P oil) had pharmacological activity. They decreased the proliferation of the prostate cells and intercepted the number of smooth muscle cells (SMCs) in the prostate, bladder, bladder neck, and supporting vasculature. Moreover, significant (*p* < .05) decreases in the prostate weight and prostate index by 54.05% and 45.81%, respectively, were noted in the treatment groups that were given transdermal optimized TM-S/P-NLC compared with the groups of animals treated with the TM-NLC, S/P-NLC, or testosterone-only formulations. Additionally, the optimized TM-S/P-NLC also exhibited remarkably improved results as compared with the oral TM tablets. Therefore, the results indicated synergistic activity of the TM-S/P-NLC when TM and S/P oils were combined in the same nanoformulation.

**Table 5.** Effect of different formulations on body weight, prostate weight, and prostate index in testosterone-induced BPH in rats.

Group	Rat weight	Prostate weight	Prostate index (×10^3^)
Group A (normal)	275.1 ± 13.2	0.71 ± 0.03	2.58 ± 0.12
Group B (testosterone only)	284.5 ± 18.5	1.14 ± 0.07	4.01 ± 0.29
Group C (optimized TM-S/P-NLCs)	268.4 ± 10.9	0.74 ± 0.02	2.75 ± 0.10
Group D (optimized TM-NLCs prepared without S/P oil)	271.3 ± 15.1	0.89 ± 0.03	3.28 ± 0.11
Group E (optimized S/P-NLCs prepared without TM)	260.2 ± 13.3	0.92 ± 0.05	3.53 ± 0.21
Group F (commercially available TM tablet)	271.8 ± 21.0	0.98 ± 0.04	3.60 ± 0.21

As shown in Table 6, the nonsignificant difference (*p >* .05) between the normal control group (Group A) and Group C, which was treated with the optimized TM-S/P-NLC, indicated the efficient activity of the optimized nanoformulation against testosterone-induced BPH. Further, when the group given the optimized TM-S/P-NLC (Group C) was compared with the group given testosterone only (Group B), group given the optimized TM-NLC prepared without S/P oil (Group D), group given the optimized S/P-NLC prepared without TM (Group E), and group given the commercially available TM tablet (Group F), a significant difference was observed (*p* < .01) in favor of the TM-S/P-NLC, and this validated the finding that the combination of TM with the S/P oils in the NLC led to synergistic activity against BPH. Thus, the result of in vivo study testified to the outcomes of in vitro release and pharmacokinetic studies. The therapeutic efficacy of optimized TM-S/P-NLC was achieved due to its better release and pharmacokinetics, which carried the drug molecules to the diseased area in the desired quantity. Whereas other formulations (optimized TM-NLCs prepared without S/P oil, optimized S/P-NLCs prepared without TM, commercially available TM tablet) were found unable to carry the drug to the diseased area in the required quantity, so multiple-dose was needed to achieve the therapeutic efficacy of TM. In addition, TM-S/P-NLC demonstrated more remarkable results against BPH than other formulations. Therefore, it is said that the results of in vitro studies highlight the in vivo characteristics of the developed formulation.

**Table 6.** Effect of various formulations shown by Tukey honest significant difference (HSD) test results in testosterone-induced BPH in rats.

Pairs of treatment	Tukey HSD Q statistic	Tukey HSD (*p*-value)	Tukey HSD inference
A vs B	30.8647	0.0010053	*p* < 0.01
A vs C	2.4019	0.5380962	Insignificant
A vs D	13.3307	0.0010053	*p* < .01
A vs E	15.0120	0.0010053	*p* < .01
A vs F	20.0561	0.0010053	*p* < .01
B vs C	28.4628	0.0010053	*p* < .01
B vs D	17.5340	0.0010053	*p* < .01
B vs E	15.8527	0.0010053	*p* < .01
B vs F	10.8087	0.0010053	*p* < .01
C vs D	10.9287	0.0010053	*p* < .01
C vs E	12.6101	0.0010053	*p* < .01
C vs F	17.6541	0.0010053	*p* < .01
D vs E	1.6813	0.8185714	Insignificant
D vs F	6.7254	0.0010053	*p* < .01
E vs F	5.0440	0.0142407	*p* < .05

## 4. Conclusion

The current study was performed to improve the bioavailability of TM and its therapeutic activity against BPH with the help of an S/P oil mixture. In this case, various TM-S/P-NLCs were prepared using experimental design-based CCD, and an optimized TM-S/P-NLC was obtained after the statistical analysis of various selected responses, such as particle size, EE, and steady-state flux. The optimized TM-S/P-NLC had a particle size, EE, and flux of 102 nm, 65%, and 4.5 μg/cm^2^.min, respectively. Additionally, stability studies showed that the optimized TM-S/P-NLC was stable in the given thermodynamic conditions. The drug release study and pharmacokinetic studies demonstrated outcomes in favor of the optimized TM-S/P-NLC. Simultaneously, in vivo therapeutic efficacy studies showed significantly favorable results for the TM-S/P-NLC in terms of prostate weight and prostate index in BPH treatment. Thus, the findings of this study showed the novelty of the developed TM-S/P-NLC in BPH treatment. The optimal TM-S/P-NLC could be a novel drug delivery system for treating BPH in the future.

## Data Availability

All data available are reported in the article.
